# TGFbeta Family Members Are Key Mediators in the Induction of Myofibroblast Phenotype of Human Adipose Tissue Progenitor Cells by Macrophages

**DOI:** 10.1371/journal.pone.0031274

**Published:** 2012-02-15

**Authors:** Virginie Bourlier, Coralie Sengenès, Alexia Zakaroff-Girard, Pauline Decaunes, Brigitte Wdziekonski, Jean Galitzky, Phi Villageois, David Esteve, Patrick Chiotasso, Christian Dani, Anne Bouloumié

**Affiliations:** 1 UMR1048, Institut National de la Santé et de la Recherche Médicale (INSERM), Université Toulouse III Paul-Sabatier, Institut des Maladies Métaboliques et Cardiovasculaires (I2MC), Toulouse, France; 2 UMR6543, Centre National de la Recherche Scientifique (CNRS), Université de Nice Sophia-Antipolis, Faculté de Médecine, Nice, France; 3 Hopital Purpan, Service Chirurgie Digestive, Toulouse, France; Centro Cardiologico Monzino, Italy

## Abstract

**Objective:**

The present study was undertaken to characterize the remodeling phenotype of human adipose tissue (AT) macrophages (ATM) and to analyze their paracrine effects on AT progenitor cells.

**Research Design and Methods:**

The phenotype of ATM, immunoselected from subcutaneous (Sc) AT originating from subjects with wide range of body mass index and from paired biopsies of Sc and omental (Om) AT from obese subjects, was studied by gene expression analysis in the native and activated states. The paracrine effects of ScATM on the phenotype of human ScAT progenitor cells (CD34^+^CD31^−^) were investigated.

**Results:**

Two main ATM phenotypes were distinguished based on gene expression profiles. For ScAT-derived ATM, obesity and adipocyte-derived factors favored a pro-fibrotic/remodeling phenotype whereas the OmAT location and hypoxic culture conditions favored a pro-angiogenic phenotype. Treatment of native human ScAT progenitor cells with ScATM-conditioned media induced the appearance of myofibroblast-like cells as shown by expression of both α-SMA and the transcription factor *SNAIL*, an effect mimicked by TGFβ1 and activinA. Immunohistochemical analyses showed the presence of double positive α-SMA and CD34 cells in the stroma of human ScAT. Moreover, the mRNA levels of *SNAIL* and *SLUG* in ScAT progenitor cells were higher in obese compared with lean subjects.

**Conclusions:**

Human ATM exhibit distinct pro-angiogenic and matrix remodeling/fibrotic phenotypes according to the adiposity and the location of AT, that may be related to AT microenvironment including hypoxia and adipokines. Moreover, human ScAT progenitor cells have been identified as target cells for ScATM-derived TGFβ and as a potential source of fibrosis through their induction of myofibroblast-like cells.

## Introduction

Excessive development of adipose tissue (AT) in obesity is characterized by an accumulation of immune cells [1]–[4]. In several models of murine obesity, the dynamic phase of AT growth is associated with monocyte recruitment. Adipose tissue macrophages (ATM) originating from these newly recruited monocytes showed a marked inflammatory phenotype in comparison to resident ATM. Such a subset has been involved in the establishment of the systemic low grade inflammation seen in obesity and insulin resistance [1], [2], [4]–[6]. Much less is known about the origin, phenotypes and function of macrophages in human AT. Human ATM (hATM) have been described to be less polarized, which may be considered as an index of chronic inflammation [7]–[9]. Indeed, hATM expressed both pro- and anti-inflammatory markers and the lymphatic vessel endothelial hyaluronan receptor 1 (LYVE-1), a specific marker of macrophages involved in tumor growth and wound healing, as well as in mouse AT angiogenesis [10], [11]. In addition, hATM-conditioned media stimulated AT-derived endothelial cell migration and organization in capillary-like structures [8]. Interestingly, hATM also expressed and secreted matrix metalloproteinase 9 (MMP-9) [8]. MMP-9 is not only a key enzyme involved in angiogenesis [8], [12] but is also responsible for the proteolytic activation of latent transforming growth factor beta (TGFβ) [13], itself implicated in the development of fibrosis [14]. Indeed, TGFβ is known to induce the appearance of extracellular matrix-secreting myofibroblasts, *via* the enhanced expression of several developmental transcription factors, including Snail and Slug [15], [16]. In obese mice models, Strissel *et al*. reported, a widespread deposition of collagen that coincided with adipocyte death and macrophage infiltration [17]. More recently, data in humans confirmed that fat mass extension was correlated with collagen deposition and fibrosis within AT, leading to systemic metabolic disturbances [18]–[20]. The number of ATM and their phenotype and location within the AT appeared to be closely related to the foci of fibrosis [20]. Interestingly, large scale transcriptomic analyses of AT from obese humans, together with immunohistochemical analysis and *in vitro* approaches, have shown that inflammatory (i.e, LPS-stimulated) monocyte-derived macrophages induced phenotypic alterations of human AT progenitor cells that resulted in excessive synthesis of extracellular matrix components [21], [22]. Furthermore, we and others have shown that factors secreted by hATM inhibited the adipogenesis of human AT progenitor cells either directly or through the enhanced expression of a TGFβ family member, *INBHA*/activinA [8], [23], [24]. The fate of these AT progenitor cells arrested by hATM-derived factors remains to be established.

The present study was undertaken to 1) investigate the influence of adiposity, AT location (subcutaneous *versus* omental) and its microenvironment on the phenotype of native hATM in terms of angiogenic and matrix remodeling/pro-fibrotic factors and 2) analyze the phenotype of native human AT progenitor cells arrested by macrophage-derived factors.

## Materials and Methods

### Materials

Chemicals were from Sigma (Saint-Quentin Fallavier, France). Collagenase type 1 was from Worthington Biochemical Corporation (Lakewood, USA). Selection kits for CD34^+^ and CD14^+^ cells were from StemCell Technologies (Grenoble, France). Culture media were from Promocell (Heidelberg, Germany). Primary antibodies for immunochemistry were from Sigma (mouse monoclonal anti-α-SMA/clone 1A4, Saint-Quentin Fallavier, France), Epitomics (rabbit monoclonal anti-CD34, Euromedex, Mundolsheimm, France), R&D Systems (mouse monoclonal anti-TGFβ (clone 1D11) and anti-activinA, Lille, France) and Dako (mouse IgG1 and mouse monoclonal anti-smooth muscle myosin heavy chain (clone SMM-1), Trappes, France) and the secondary antibodies from Invitrogen (Cergy-Pontoise, France).

### Preparation of human AT stroma vascular fraction (SVF) and mature adipocytes

Human subcutaneous (Sc) AT were obtained from healthy women, undergoing elective surgery for fat removal for aesthetic purposes (mean age 43.8±1.3 years, mean body mass index (BMI) 27.7±0.6 kg/m^2^). Human omental (Om) and subcutaneous (Sc) AT were obtained from a group of obese non-diabetic patients undergoing bariatric surgery (22 paired biopsies; 20 women/2 men; mean age 41.3±2.5 years (from 26 to 61 years), mean BMI 43.9±1.4 kg/m^2^ (from 37 to 59 kg/m^2^), and mean waist-circumference 124.5±2.7 cm (from 109 to 151 cm). All patients gave their informed consent. Based on medical information, the mean time since obesity has been diagnosed was 19.7±2.3 years, their weights were stable for at least three months before surgery and 1 patient was identified as pre-diabetic, 3 had hypercholesterolemia, 1 triglyceridemia and 6 patients were hypertensive. The protocol for this study was approved by the Institutional Research Board of INSERM and Toulouse University Hospital. The AT was immediately processed after removal and digested using collagenase (250 U/mL in phosphate-buffered saline (PBS), 2% bovine serum albumin (BSA), pH 7.4, volume/volume) for 30 min at 37°C under constant shaking. After a brief centrifugation (100 g, 30 s, room temperature (RT)), adipocytes were recovered and washed in order to be either plated in fibrin gels [3] with basal medium (i.e. Endothelial Cell Basal Medium (ECBM)/0.1% BSA/0.05 mg/ml gentamycin) for 24 h to collect conditioned media (CM), or lyzed in Qiazol lysis reagent (volume/volume, Qiagen, Courtaboeuf, France) for lipid and RNA extraction. Adipocyte-CM were collected, centrifuged (20 000 g, 3 min, RT) and stored at −20°C until further use. After another centrifugation (300 g, 10 min, RT), the pellet containing the SVF was resuspended in erythrocyte lysis buffer (155 mmol/L NH_4_Cl; 5.7 mmol/L K_2_HPO_4_; 0.1 mmol/L EDTA (ethylenediaminetetraacid); pH 7.3) for 10 min. Finally, and after successive filtrations through 100-, 70- and 40- µm sieves, the SVF cells were resuspended in PBS/2% fetal calf serum (FCS) and used to separate the different cell types.

### Isolation and culture conditions of the cells from human AT SVF

CD34^+^/CD31^−^, defined as AT progenitors cells, CD34^+^/CD31^+^, defined as endothelial cells, and CD34^−^/CD14^+^ cells, defined as ATM, were isolated from ScAT SVF using an immunoselection/depletion protocol as previously described [3], [25], [26].

Freshly isolated CD34^−^/CD14^+^ cells (i.e, ATM), were either then lyzed in RLT lysis buffer (Qiagen, Courtaboeuf, France) and stored at −20°C for further mRNA extraction or cultured (250 000 cells/cm^2^) for 24 h (i) in basal medium under a normoxic or hypoxic (1% O_2_) atmosphere or (ii) treated with mature adipocyte-CM. Normoxic 24 h-basal media conditioned by ATM were collected, centrifuged (20 000 g, 3 min, RT) and stored at −20°C until further use.

Freshly isolated CD34^+^/CD31^−^ AT progenitors cells were either lyzed in RLT lysis buffer (Qiagen, Courtaboeuf, France) and stored at −20°C for further mRNA extraction or cultured (10 000 cells/cm^2^) in ECBM/10%FCS until confluence. AT progenitor cells were then trypsinized (TrypLE Express, Gibco/Invitrogen, Cergy Pontoise, France), plated at 80 000 cells/cm^2^ and left for a few hours to recover. Prior to use, progenitor cells were washed to eliminate FCS and left overnight in basal medium. Medium was then replaced for 24 h or 48 h (gene expression analysis) or 48 h (immunocytochemistry) by (i) control basal medium, (ii) ATM-CM, (iii) basal medium containing human recombinant TGFβ1 (5 ng/ml, eBioscience, Paris, France) or (iv) basal medium containing human recombinant activinA (100 ng/ml, Peprotech, Neuilly sur Seine, France).

Freshly isolated CD34^+^/CD31^+^ endothelial cells were lysed in RLT lysis buffer and stored at −20°C for further mRNA extraction.

### Human multipotent adipose-derived stem (hMADS) cell culture and activinA treatment

hMADS cells were obtained from the stroma of human AT as described previously [27]. The cells were isolated from the pubic region fat pad of a 4-month old (hMADS3) male donor. Cells were plated at 22 000 cell/cm^2^ in proliferation medium (DMEM (low glucose), 10% FCS, 100 U/ml penicillin and streptomycin). After 24 h, the proliferation medium was removed and the cells maintained in DMEM containing insulin (5 µg/ml), and transferrin (10 µg/ml), supplemented or not with 100 ng activinA/ml (Peprotech). RNAs were extracted after a further 24 h and 48 h.

### RNA extraction and real-time PCR

Total RNA was extracted from AT-derived ATM, endothelial cells, progenitor cells and mature adipocytes using an RNeasy kit (Qiagen, Courtaboeuf, France). The RNA concentration was determined fluorimetrically (Ribogreen, Invitrogen, Cergy-Pontoise, France) and the RNA was reverse-transcribed using the “Superscript II” kit (Invitrogen, Cergy-Pontoise, France). Reverse transcription was also carried out on RNA samples without the superscript enzyme to ensure the absence of contaminating genomic DNA. Primers for interleukin 6 (IL-6), interleukin 10 (IL-10), transforming growth factor β (TGFβ1), monocyte chemotactic protein 1 (MCP-1), matrix metalloproteinase 2 (MMP-2), matrix metalloproteinase 9 (MMP-9), lymphatic vessel endothelial hyaluronan receptor 1 (LYVE-1), hypoxia-inducible factor 1α (HIF-1α), hypoxia-inducible factor 2α (HIF-2α/EPAS1), vascular endothelial growth factor (VEGFA), Snail (snail1), Slug (snail2), inhibinA (activinA), TGFβ receptor 1 (ALK-5), Smad2, Smad3, fibronectin (fibronectin 1), activinA receptor type I (ACVR1A/ALK-2) and plasminogen activator inhibitor-1 (PAI-1) were from Applied Biosystems (Courtaboeuf, France) (Hs00174131_m1, Hs00174086_m1, Hs00171257_m1, Hs00234140_m1, Hs00234422_m1, Hs00234579_m1, Hs00272659_m1, Hs00156153_m1, Hs00181674_m1, Hs00173626_m1, Hs00195591_m1, Hs00161904_m1, Hs00170103_m1, Hs00610319_m1, Hs00183425_m1, Hs00969210_m1, Hs00365058_m1, Hs00153836_m1, Hs00167155_m1, respectively). The amplification reaction was done in duplicate on 15 ng of the cDNA samples in a final volume of 20 µL in 96-well reaction plates (Applied Biosystems) in a GeneAmp 7500 detection system. All reactions were carried out under the same conditions: 50°C for 2 min, 95°C for 10 min, 40 cycles of 95°C for 15 sec and 60°C for 1 min. Results were analyzed with the GeneAmp 7500 software and all the values were normalized to the levels of 18S rRNA (Applied Biosystems, VIC/TAMRA probe, part n°4310893E).

For hMADS cells, total RNA was extracted using an RNeasy kit (Qiagen, Courtaboeuf, France) and the RT-PCR analysis was done using superscript II reverse transcriptase (Invitrogen, Cergy-Pontoise, France) according to the manufacturer's instructions. Real-time PCR assays were run on an ABI Prism 7000 real-time PCR machine (PerkinElmer Life Sciences). Normalization was done using the geometric averages of the housekeeping genes, G6PDH (glucose-6-phosphate dehydrogenase), POLR2A (polymerase RNA II), and TBP (TATA box binding protein) and quantification using the comparative-ΔCt method. The primer sequences used for quantitative PCR are detailed in Table S1.

### Immunocytochemistry and immunohistochemistry

AT progenitor cells treated for 48 h with TGFβ1, ATM-CM or ATM-CM plus neutralizing antibodies (1 h pre-incubation of ATM-CM with anti-TGFβ 1 µg/mL, anti-activinA 1 µg/mL or both) were fixed (30 min, paraformaldehyde 4%, RT), washed and incubated for 1 h in PBS/2% BSA/0.1%Triton followed by an overnight incubation with α-smooth muscle actin (SMA) or smooth muscle myosin mouse monoclonal antibody (1/250 and 1/100 respectively). After washing (1 h, PBS/0.2% Tween), cells were incubated for 90 min with the corresponding fluorescence-labeled second antibody (goat anti-mouse coupled to AlexaFluor 546, 1/250). Cells were washed and incubated for 10 min with 10 µg/mL Hoechst 33242 (Invitrogen, Cergy-Pontoise, France) to stain nuclei and washed again before direct observation with a fluorescence microscope (Nikon) and representative images were recorded (NIS-Elements BR software). Mouse monoclonal IgG1 was used as a negative control. The number of α-SMA^+^ foci in the whole well (total magnification ×20) was counted for analysis. To analyze the effects of neutralizing antibodies, α-SMA^+^ foci were counted on 3 to 4 standardized fields (total magnification ×10) and normalized to nuclei number (Image J 1.44p software, NIH, USA).

Pieces of ScAT (2–3 mm^3^) were fixed in neutral buffered 4% (w/v) paraformaldehyde (1 h, RT), blocked in PBS/3% BSA (30 min, RT), and incubated in PBS/0.1% BSA/0.2% Triton/0.05% Tween with primary antibodies overnight at 4°C (i.e, CD34 1/100 and α-SMA 1/80). After washing, the ScAT samples were incubated for 1 h with secondary antibodies (goat anti-mouse or goat anti-rabbit coupled to AlexaFluor 546 or 488, 1/250). Nuclei were stained with Hoechst 33242. Fluorescence analysis was carried out utilizing a Nikon inverted Eclipse TE300 microscope and NIS-Elements BR software.

### Statistical analysis

Values are given as mean ± SE mean for (n) separate experiments. Comparisons between groups were analyzed either by unpaired (Mann-Whitney) or paired (Wilcoxon or Student) tests or one-way ANOVA (parametric or non parametric, for repeated measure or not), followed by a Dunn's multiple comparison test, respectively (Prism 4, GraphPad Software, USA). Differences were considered significant when P<0.05.

## Results

### Impact of obesity and AT location on the remodeling phenotypes of native human AT macrophages

**Figure 1:**
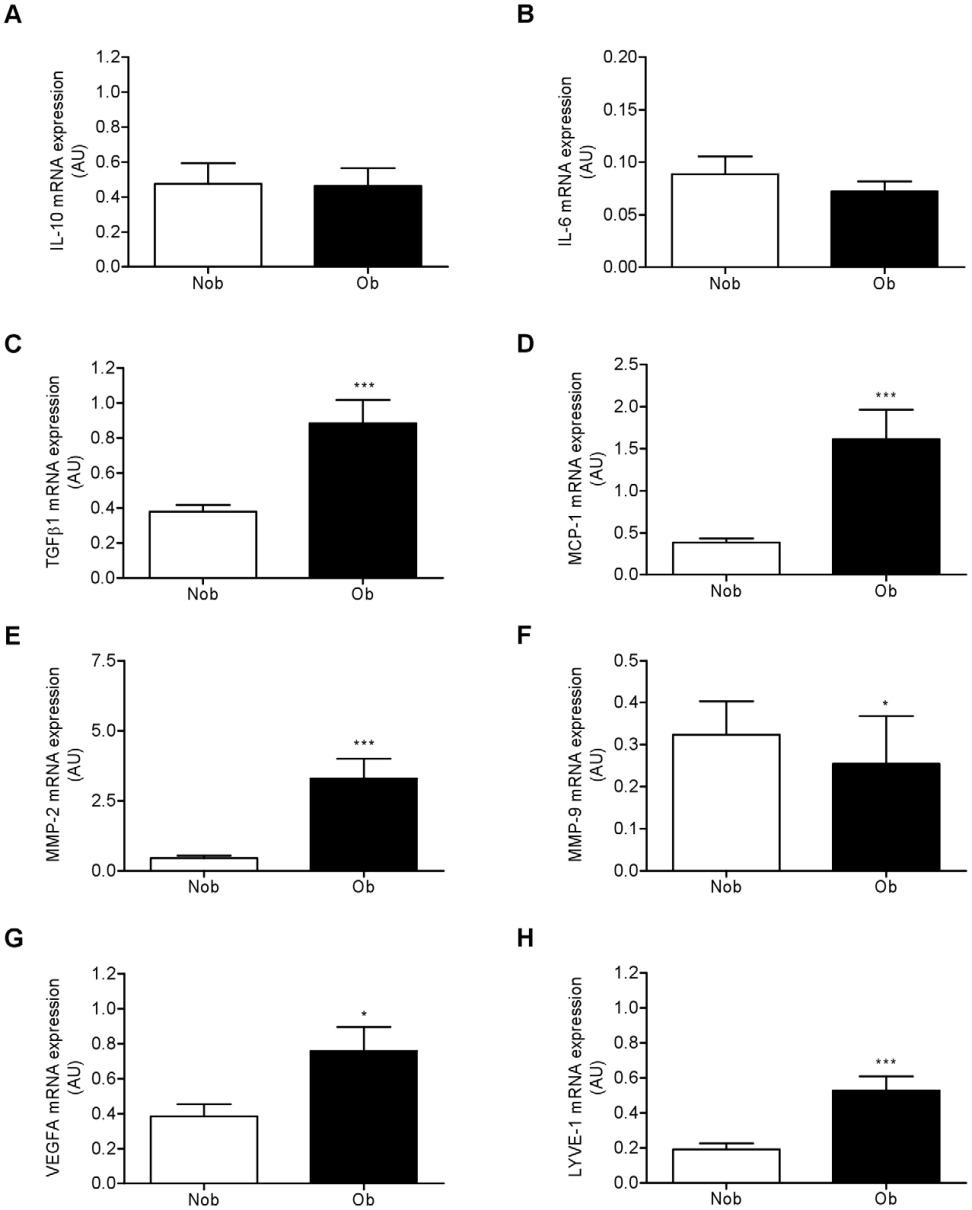
Obesity status affects the remodeling phenotype of human subcutaneous AT macrophages. ATM were immunoselected from subcutaneous AT from 18 non obese individuals (Nob, mean BMI 25.4±0.6 kg/m^2^) and 22 obese individuals (Ob, mean BMI 43.9±1.4 kg/m^2^) and the transcript levels of angiogenic and matrix remodeling/fibrotic factors were determined by real-time PCR analyses. Values are means ± SEM (AU, arbitrary unit). * P<0.05, *** P<0.001 *vs* Nob. IL, interleukin; MCP-1, monocyte chemotactic protein 1; MMP, matrix metalloproteinase; VEGF, vascular endothelial growth factor; LYVE-1, lymphatic vessel endothelial hyaluronan receptor 1.

**Figure 2:**
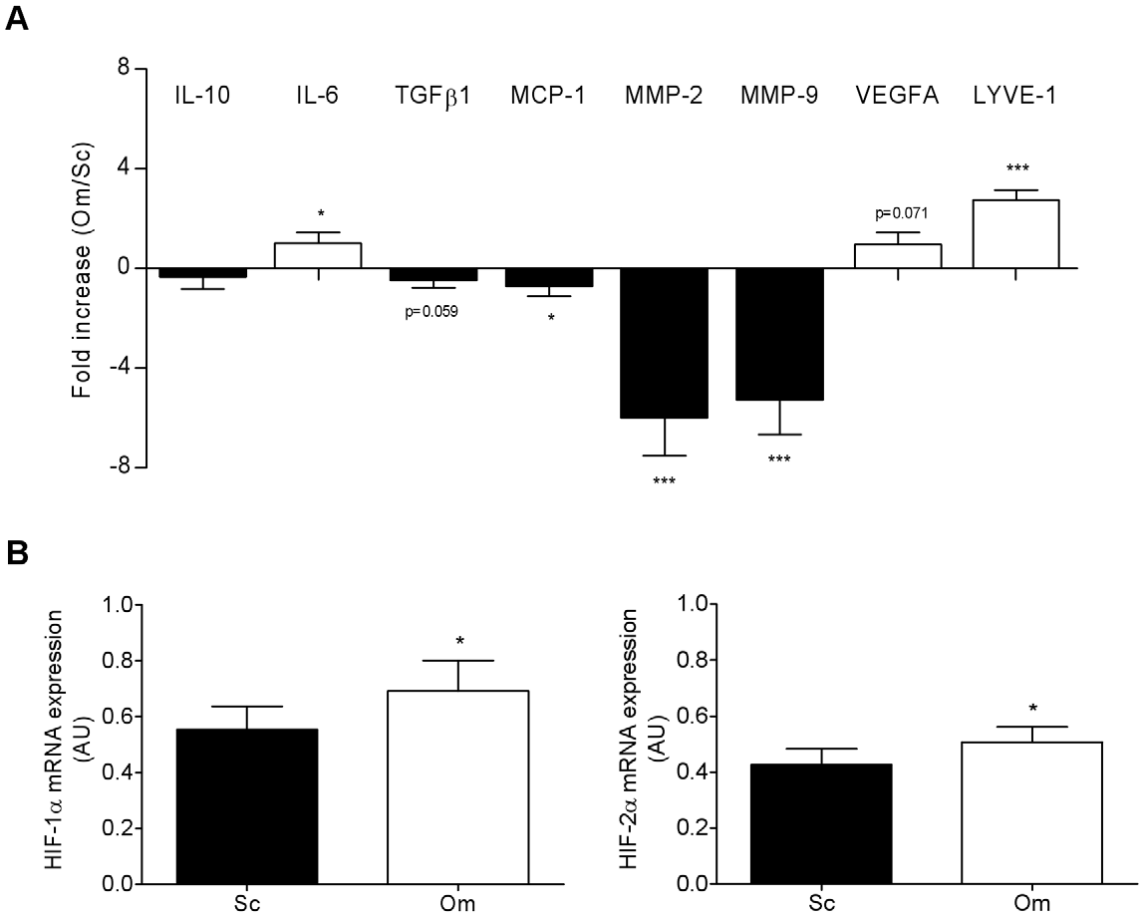
AT location affects the remodeling phenotype of human AT macrophages in obese individuals. A, Transcript levels of angiogenic and matrix remodeling/fibrotic factors were determined by real-time PCR analyses of ATM immunoselected from paired biopsies of subcutaneous (Sc) and omental AT (Om). Results are expressed as fold differences between Om and Sc and are means ± SEM (n = 22 subjects, mean BMI 43.9±1.4 kg/m^2^). Open bars: genes up-regulated, and solid bars: genes down-regulated, in OmATM *vs* ScATM. B, Transcript levels of *HIF-1α* and *-2α* determined by real-time PCR analyses of ScATM and OmATM. Values are means ± SEM (AU, arbitrary unit) of the 22 paired biopsies. * P<0.05 and *** P<0.001 *vs* Om.

The expression of several genes involved in angiogenesis and matrix remodeling/fibrosis (*IL-10*, *IL-6*, *TGFβ1*, *MCP-1*, *MMP-2* and *-9*, *VEGFA* and *LYVE-1*) were analyzed by real-time PCR in human native ATM (CD34^−^/CD14^+^) immunoselected from the ScAT of non-obese and obese individuals. As shown in the figure 1, the levels of expression of *TGFβ1* as well as *MCP-1*, *MMP-2*, *LYVE-1* and *VEGFA* were markedly higher whereas *MMP-9* transcript levels were lower in the ATM isolated from the obese compared to the non-obese group. Similar analyses on native ATM isolated from paired ScAT and OmAT biopsies of obese individuals showed that OmATM expressed more angiogenic markers than ScATM as shown by the higher levels of *VEGFA*, *LYVE-1* and *IL-6* (Fig. 2A). In contrast, ScATM showed higher expression of factors involved in matrix remodeling and fibrosis such as *TGFβ1* as well as *MCP-1* and *MMPs* (2 and 9). Higher levels of both *HIF-1α* and *HIF-2α* transcripts were observed in OmATM versus ScATM (Fig. 2B).

### Impact of hypoxia and adipokines on the remodeling phenotypes of native human AT macrophages

**Figure 3:**
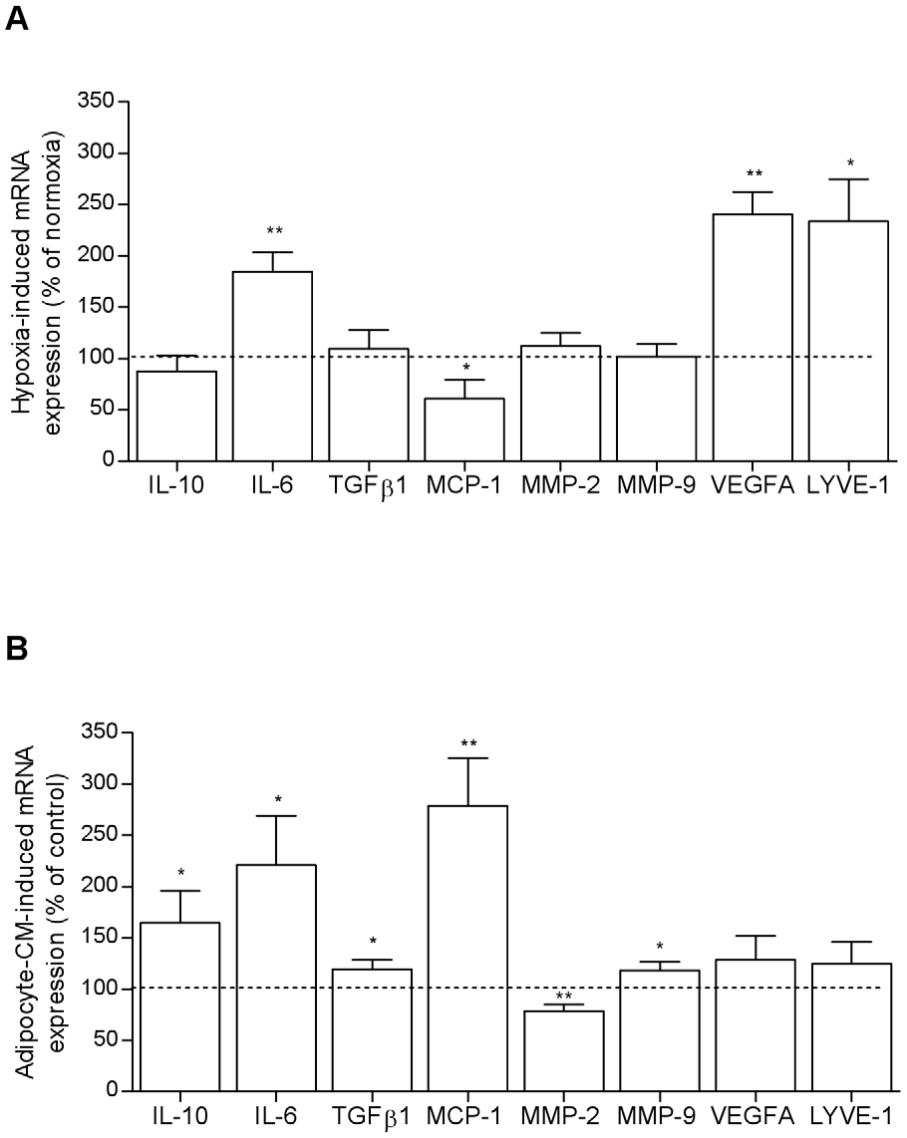
Hypoxia and mature adipocyte-derived factors affect the remodeling phenotype of human subcutaneous AT macrophages. Transcript levels of angiogenic and matrix remodeling/fibrotic factors were determined by real-time PCR analyses on ScATM cultured for 24 h (A) in hypoxic conditions (1% O_2_, n = 5) and (B) in mature subcutaneous adipocyte-conditioned media (CM, n = 5). Values, expressed as a percentage of the control, are means ± SEM. * P<0.05 and ** P<0.01 *vs* control conditions.

Human ScATM were maintained under normoxic or hypoxic culture conditions (1% O_2_) or in the presence or absence of conditioned media from human ScAT mature adipocyte (adipocyte-CM). Real-time PCR analysis revealed distinct gene regulation profiles depending on the culture conditions. The levels of expression of both angiogenic markers *VEGFA* and *LYVE-1* were up-regulated under low oxygen tension whereas those of the factors involved in matrix remodeling and fibrosis including *TGFβ1* but also *MCP-1* and *MMP-9* were up-regulated by adipocyte-derived secretions (Fig. 3A and 3B). To note, *MMP-2* expression was found to be decreased in this condition. The expression level of *IL-6* was increased in both culture conditions whereas the one of *IL-10* was up-regulated with only adipocyte-CM.

### The human native AT progenitor cells: a cell target of TGFβ1

**Figure 4:**
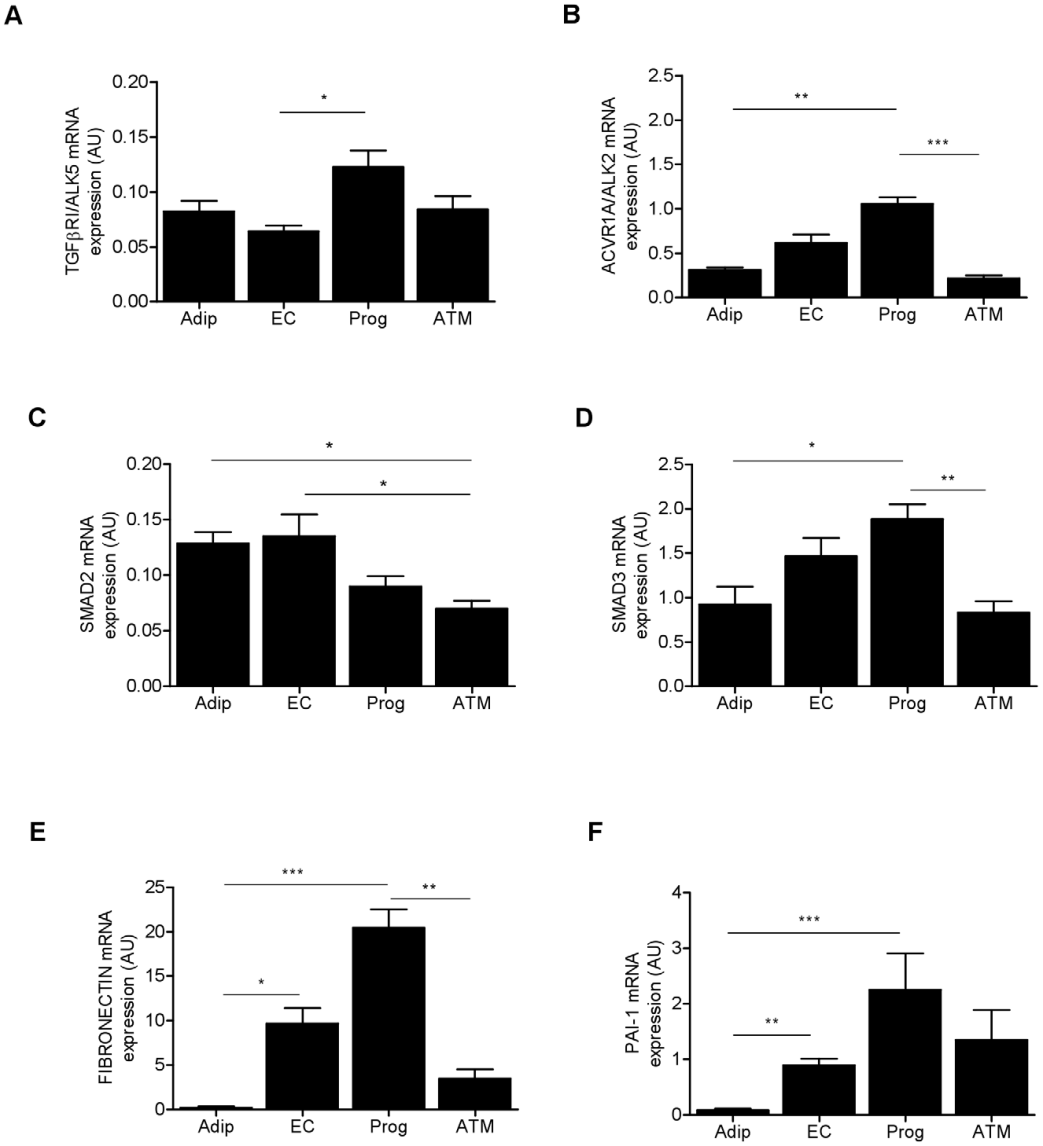
Components of TGFβ-signaling pathway in human subcutaneous AT cells. *TGFβ R1* (*ALK5*) (A), *activinA R1* (*ACVR1A/ALK2*) (B), *SMAD2* (C), *SMAD3* (D), *FIBRONECTIN* (E) and *PAI-1* (F) transcript levels were determined by real-time PCR in ScAT mature adipocytes (Adip), endothelial cells (EC), progenitor cells (Prog) and ATM. Values are means ± SEM (AU, arbitrary units) of 5 to 27 independent. * P<0.05, ** P<0.01 and *** P<0.001 between cell types.

The expression of several key components of the TGFβ signaling pathway, including TGFβ receptor 1 (*TGFβRI/ALK5*), activinA receptor type I (*ACVR1A/ALK2*), *SMAD2*, *SMAD3*, *FIBRONECTIN* and *PAI-1*, were analyzed by real-time PCR on isolated human ScAT native mature adipocytes, endothelial cells (CD34^+^/CD31^+^), progenitor cells (CD34^+^/CD31^−^) and ATM (CD34^−^/CD14^+^). As shown in figure 4, *TGFβRI* as well as *ACVR1*, *SMAD3*, *FIBRONECTIN* and *PAI-1* transcript levels were higher in native human AT progenitor cells compared with mature adipocytes, endothelial cells and/or ATM.

### Induction of a myofibroblast-like phenotype in human native AT progenitor cells by ATM-conditioned media and TGFβ family members

**Figure 5:**
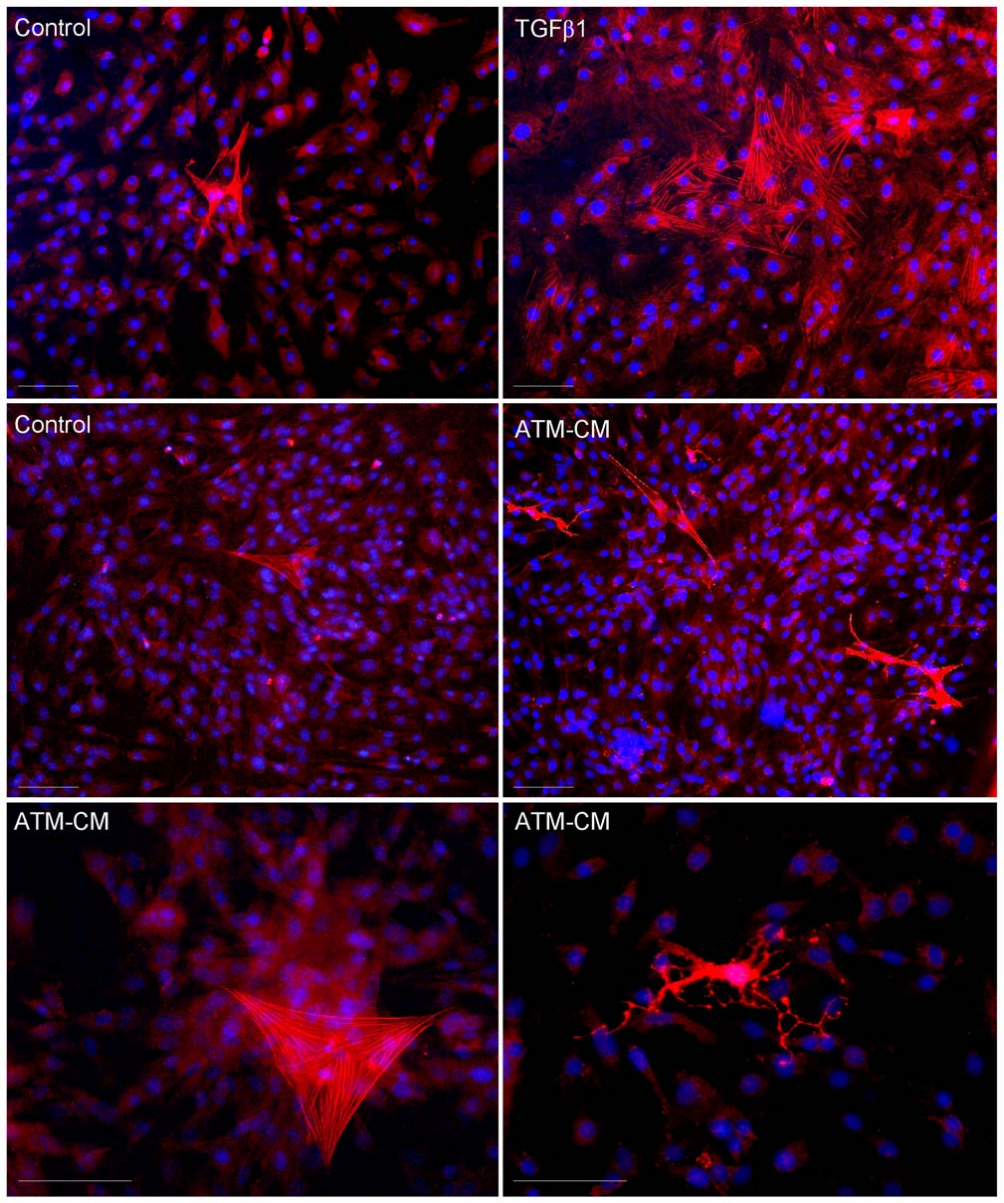
TGFβ1 and subcutaneous AT macrophage-conditioned media induce α-SMA expression in human AT progenitor cells. Representative photomicrograph of immunocytochemical staining for α-SMA (red) of ScAT progenitors cells (n = 4) cultured for 48 h with basal medium (i.e, control), with TGFβ1 (5 ng/ml, n = 4, upper panel) or with ScATM-CM (n = 6, middle and lower panels). Nuclei were stained with Hoechst 33242 (blue). Magnification ×20 and ×40. White scale corresponds to 50 µm.

**Figure 6:**
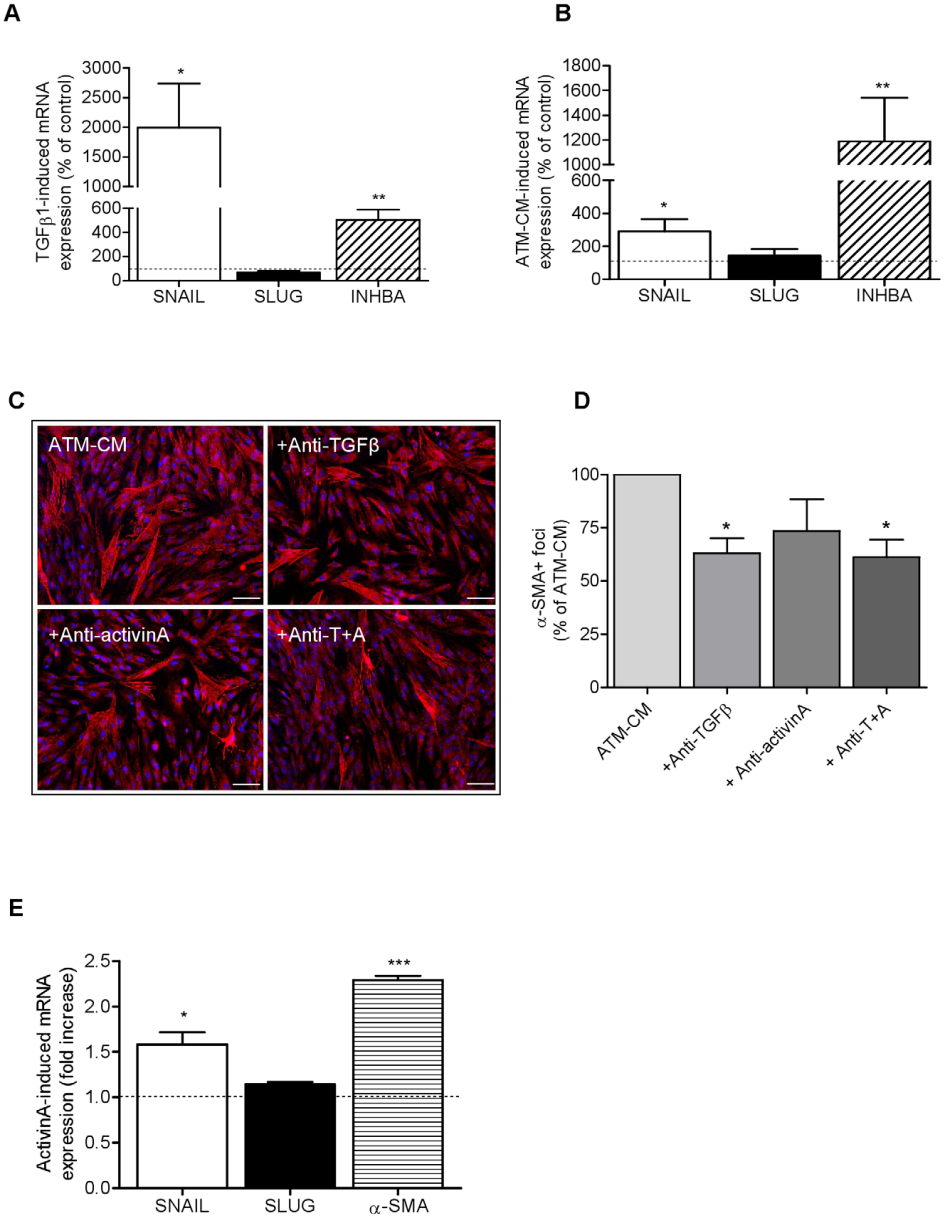
TGFβ1 and subcutaneous AT macrophage-conditioned media induce a myofibroblast-like phenotype in human AT progenitor cells. A and B, Transcript levels of *SNAIL*, *SLUG* and *INHBA*/activinA were determined by real-time PCR analyses in ScAT progenitor cells treated for 24 h with basal medium (i.e, control, n = 4 to 5), with TGFβ1 (5 ng/ml, n = 4) or with ScATM-CM (n = 6). Results are expressed as percentage of the control and are means ± SEM. * P<0.05 and ** P<0.01 *vs* control media. C, Representative photomicrograph of immunocytochemical staining for α-SMA (magnification ×10) of ScAT progenitors cells (n = 3) that were cultured for 48 h with ScATM-CM (n = 5) in the presence or not of neutralizing antibodies directed against TGFβ (1 µg/ml), activinA (1 µg/ml) or both (A+T, 1 µg/ml each). White scale corresponds to 50 µm. D, Number of α-SMA^+^ foci per 100 nuclei. Values are expressed as a percentage of ScATM-CM and are means ± SEM. * P<0.05 *vs* ScATM-CM, n = 3. E, Transcript levels of *SNAIL*, *SLUG* and *α-SMA* were determined by real-time PCR analyses in hMADS treated or not with activinA (100 ng/ml) for 24 h (n = 4). Values are expressed as fold increase of the controland are means ± SEM. * P<0.05 and *** P<0.001 *vs* control media.

ScAT progenitor cells were treated either with ScATM-conditioned media (ScATM-CM) or TGFβ1 for 48 h and the number of cells expressing the myofibroblast marker α-SMA was analyzed by immunocytochemistry. As shown in figure 5, under control culture conditions, the vast majority of ScAT progenitor cells exhibited a diffuse α-SMA labelling and few cells containing specific stress fibers were identified (25±4 α-SMA^+^ foci/well). Treatment with TGFβ1 markedly increased α-SMA^+^ stress fiber foci with no changes in total cell number (estimated by nuclei counts, data not shown). ATM-CM treatment was also associated with an increase in α-SMA^+^ stress fiber foci, although with a lower extent (58±14 α-SMA^+^ foci/well) with no changes in total cell number (data not shown). Interestingly, ATM secretions also induced the appearance of stellate-shaped myofibroblast (right panel), a distinct morphological state of myofibroblasts. In parallel, the expression of myofibroblast transcription factors, *SNAIL* and *SLUG*, were analyzed by real-time PCR as well as the expression of *INHBA/*activinA, a member of TGFβ family. As shown in figures 6A and 6B, treatment of ScAT progenitor cells with either TGFβ1 or ScATM-CM markedly up-regulated *SNAIL* and *INHBA/*activinA transcripts but had no effect on *SLUG* expression. Furthermore, treatment of native ScAT progenitor cells with activinA up-regulated *SNAIL* expression after 24 or 48 h of treatment (3.2 fold increase, n = 3, P<0.05) suggesting that the up-regulation of ActivinA expression by TGFβ treatment may contribute to the acquisition of the myofibroblast-like phenotype by AT progenitor cells. Indeed, treatment of AT progenitor cells with ScATM-CM in the presence of neutralizing antibodies directed against TGFβ1 and ActivinA, led to an inhibition of ScATM-CM effect on the number of α-SMA^+^ foci (Fig. 6C and D) although the inhibitory effect was more marked with the anti-TGFβ antibodies (3.7 fold decrease, n = 3, P<0.05) compared to activinA antibodies. To rule out the possibility of contaminations with smooth muscle cells, immunocytochemistry assays using anti-smooth muscle myosin antibody on ATM-CM treated AT progenitor cells was performed and produced no detectable signal (n = 3, data not shown). Finally, to clearly establish the effect of ActivinA on AT progenitor cells, the human AT progenitor cell line, hMADS, was used. Treatment of hMADS cells with ActivinA led to a marked up-regulation of *α-SMA* expression associated with an up-regulation of *SNAIL* but no effect on *SLUG* mRNA levels (Fig. 6E).

### Evidence of an increase in AT myofibroblast-like progenitor cells with obesity

**Figure 7:**
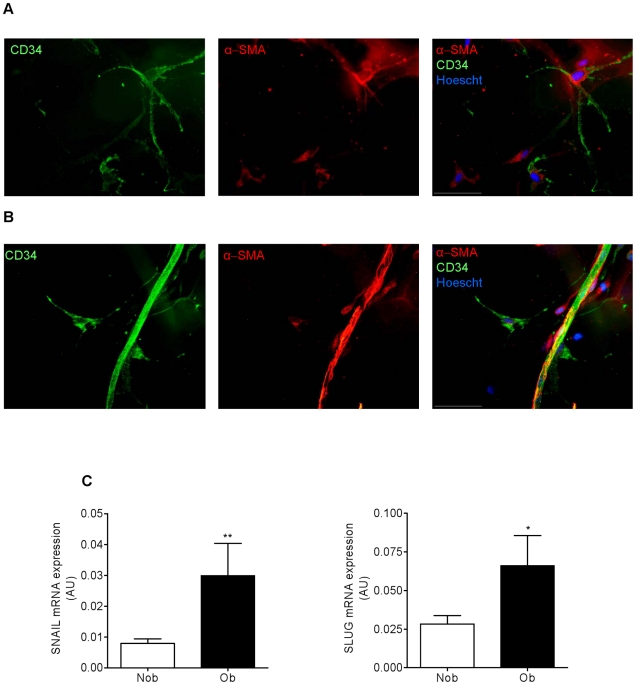
Obesity is associated with increased expression of myofibroblast markers in subcutaneous AT progenitor cells. A and B, Representative photomicrograph of immunohistochemistry of whole ScAT staining: α-SMA (red), CD34 (green) and nuclei (Hoechst 33242/blue) (n = 9). White scale corresponds to 50 µm. C, *SNAIL* and *SLUG* transcript levels were determined by real-time PCR in immunoselected ScAT progenitor cells from 7 non obese (Nob) and 8 obese (Ob) individuals. Values are means ± SEM (AU, arbitrary units). * P<0.05 and ** P<0.01 *vs* Nob.

Immunohistochemistry was carried out on human ScAT using CD34 and α-SMA antibodies. Distinct CD34^+^ as well as α-SMA^+^ cell subsets were observed. Co-labeled CD34^+^/α-SMA^+^ cells were identified in the AT stroma (Fig. 7A) whereas α-SMA^+^ but CD34^−^ cells, were found at perivascular location, most probably corresponding to pericytes (Fig. 7B). The expression of both *SNAIL* and *SLUG* were determined in native AT progenitor cells isolated from ScAT by real time PCR analyses. Both *SNAIL* and *SLUG* transcripts were found to be higher in native AT progenitor cells from obese compared with non-obese individuals (Fig. 7C).

## Discussion

Extensive AT growth has been recently associated with increased fibrosis in rodents as well as in humans [1]–[3], [17], [19], [20], [28]. We previously showed that ATM from the ScAT of lean and overweight subjects exhibited a phenotype characterized by the specific expression of *MMP-9* and pro-angiogenic properties [8]. The present study extends these data to ATM from obese individuals in agreement with recent literature [9], [29]. Moreover, based on gene expression profiles determined on native ATM, we could distinguish two main phenotypes related to angiogenesis in OmAT and to matrix remodeling/fibrosis in ScAT. Indeed, OmATM compared with ScATM from obese patients had higher transcript levels of genes involved in angiogenesis, including *IL-6*, *VEGFA* and *LYVE-1*, together with higher expression of *HIF-1α* and *HIF-2α*. Our previous study reported higher expression of hypoxia-related genes in mature adipocytes from OmAT compared with ScAT, including *HIF-1α* and *VEGFA* suggesting that hypoxia-related events are more likely to occur in OmAT than in ScAT [30]. In agreement with such a hypothesis, the present data show that hypoxic culture conditions mimicked the OmATM phenotype in ScATM, i.e. up-regulation of *IL-6*, *VEGFA* and *LYVE-1*. Interestingly, vascular network and endothelial cell number were found to be higher in OmAT versus ScAT [30]. Compared to OmATM, ScATM were characterized by higher expression of matrix remodeling/fibrosis-related genes, including *TGFβ1* as well as *MMP-2* and *-9*. The MMPs, and more particularly MMP-9, play a key role in the proteolytic activation of latent TGFβ1 [13], itself involved in extracellular matrix homeostasis [31]–[33] and fibrosis [14]. Secretions from mature subcutaneous adipocytes favored the appearance of a matrix remodeling/fibrosis-related phenotype in ScATM, as shown by the increased expression of *TGFβ1* and *MMP-9*. Unexpectedly, *MMP-2* level was decreased in this condition. Although MMP-2 and 9 are functionally related, the composition of their promoter is quite different leading to specific gene regulation [34]. Taken together, the results suggest that the location-specific and adiposity-dependent microenvironment of AT among them oxygen tension and adipokines, modulate the phenotype of ATM towards a matrix remodeling/fibrosis-related phenotype in ScAT and angiogenesis in OmAT.

Recently, total fibrosis has been described to be more abundant in ScAT versus OmAT in humans [19]. Taken into account the pivotal role of TGFβ1 in fibrosis and since TGFβ1 expression in ATM was higher in obese than non-obese on one hand and in the other in ScAT versus OmAT, we hypothesize a role of ATM in the genesis of ScAT fibrosis. First, the AT potential cell targets of TGFβ1 were analyzed. Human native AT progenitor cells (i.e, CD34^+^/CD31^−^ cells) in comparison with mature adipocytes, endothelial cells (i.e, CD34^+^/CD31^+^ cells) and ATM, expressed higher levels of the key components of the TGFβ family signaling pathway, including *TGFβR1* (*ALK5*), *ACVR1A* (*ALK2*) and *SMAD3*, as well as specific target genes including *FIBRONECTIN* and *PAI-1*. Therefore, among the distinct cell populations constituting human AT, progenitor cells appear to be the main cell target of TGFβ. TGFβ1 treatment of AT progenitor cells led to an increase in aSMA^+^ stress fiber foci associated with an up-regulation of both *INHBA*/activinA and the transcription factor *SNAIL*, hallmark of myofibroblast [35], [36]. Such an effect was mimicked by conditioned media from ScATM. To note, in addition to the increase in α-SMA^+^ stress fiber foci, ScATM-conditioned media treatment led to the appareance of stellate-shaped myofibroblasts. Although additional experiments will be required to clearly characterize the myofibroblasts arising from the treatment with ScATM, it has been suggested that myofibroblasts might exhibit distinct cell morphology according to their maturation and/or functions [37]. We have previously reported that CM from ScATM inhibit adipogenesis [8] and increase the expression of *INHBA*/activinA in native AT progenitor cells [24]. Treatment of native progenitor cells with activinA was associated with increased expression of *SNAIL*. Finally, when TGFβ and to a lower extent activinA were neutralized in ATM-CM, the number of α-SMA^+^ foci were reduced. To clearly establish the specificity of activinA effect on AT progenitor cells, treatments were performed on human AT progenitor cell line, hMADS, since previous results showed that ScATM treatment of hMADS led to an inhibition of adipogenesis together with an up-regulation of activinA [24]. In the present study, we show that activinA treatment of hMADS cells led to an up-regulation of *α-SMA* and *SNAIL* expression. Taken together, the present study shows that ATM-derived factors and most specifically TGFβ1 induced a myofibroblast-like phenotype of AT progenitor cells. Such an effect may be mediated, at least in part, through the induction of *INHBA*/activinA expression by the progenitor cells.

α-SMA^+^ cells were recently identified *in situ* in fibrotic foci of ScAT from obese individuals [19]. We show that among α-SMA^+^ cells present within human ScAT, some exhibited pericyte-like characteristics, ie. negative for CD34 and in perivascular location. Other α-SMA^+^ were localized in the AT stroma and co-expressed CD34, suggesting that such a cell subset might arise from AT progenitor CD34^+^/CD31^−^ cells. Gene expression analysis of native ScAT progenitor cells revealed that the expressions of both *SNAIL* and *SLUG* were higher in obese compared with lean individuals. Altogether, it is tempting to speculate that some progenitor cells in ScAT from obese individuals are induced towards a myofibroblast-like phenotype due to changes in the microenvironment, especially the pro-fibrotic TGFβ1-derived human ScATM. However, additional studies are necessary to clearly define the mechanisms involved in the induction of myofibroblast phenotype including the signaling pathway as well as the consequence of such an event.

In conclusion, the present results show that human native ATM exhibit distinct phenotypes according to the location of the AT and the degree of adiposity. These phenotypes might be linked to the AT microenvironment including adipokines and hypoxia. In human ScAT, ATM-derived secretions, including TGFβ1, promote the appearance of a myofibroblast-like phenotype of human AT progenitor cells that may be mediated through the induction of *INHBA/*activinA. In murine models, AT fibrosis has been associated with physical constrains limiting adipocyte hypertrophy [38]. Since AT native progenitor cells treated with hATM-CM reduced their adipogenic capacity [8], one might speculate that the reorientation of the human AT progenitor cells into myofibroblasts-like cells indirectly contributes to a reduction in adipocyte hyperplasia. Consequently both decreased adipocyte hypertrophy and hyperplasia leading to reduced ScAT expandability may promote ectopic fat deposition. Therefore TGFβ family members (i.e, TGFβ1 and activinA) produced by ScATM and AT progenitor cells represent interesting targets to improve the storage capacity of ScAT.

## Supporting Information

Table S1 **Primer sequences used for real-time PCR in hMADs.** ASMA, α-smooth muscle actin; G6PDH, glucose-6-phosphate dehydrogenase; POLR2A, polymerase RNA II; TBP, TATA box binding protein.
(DOC)
