# Supplementary material for: TGFbeta Family Members Are Key Mediators in the Induction of Myofibroblast Phenotype of Human Adipose Tissue Progenitor Cells by Macrophages
Source: PLoS One. 2012 Feb 15;7(2):e31274. doi: 10.1371/journal.pone.0031274 (PMC3280291; doi:10.1371/journal.pone.0031274)
Supplement: Table S1 — Primer sequences used for real-time PCR in hMADs. ASMA, α-smooth muscle actin; G6PDH, glucose-6-phosphate dehydrogenase; POLR2A, polymerase RNA II; TBP, TATA box binding protein. (DOC) [file pone.0031274.s001.doc]

Gene forward reverse

SNAIL GCTGCAGGACTCTAATCCAGAGTT GACAGAGTCCCAGATGAGCATTG

SLUG ATGAGGAATCTGGCTGCTGT CAGGAGAAAATGCCTTTGGA

ASMA TGGATCAGCAACAGAATACG GCATTTGCGGTGGACAATG

G6PDH ACCTTCGCAGCCGTCCTAGT ATCCGAGCGTAGCCCACTCT

POLR2A TGGGTGTGCCCCGACTTAA TCCAGACGGCACAGAATATCCT

TBP ACGCCAGCTTCGGAGAGTTC CAAACCGCTTGGGATTATATTCG
